# Single-cortex is better than double-cortex in fibula grafts for large tibia bone defect in a 2-year-old child: a case report of a successful surgery and discussion of bone graft choices: Erratum

**DOI:** 10.1097/MD.0000000000007809

**Published:** 2017-08-11

In the article, “Single-cortex is better than double-cortex in fibula grafts for large tibia bone defect in a 2-year-old child: A case report of a successful surgery and discussion of bone graft choices”,^[1]^ which appeared in Volume 96, Issue 5 of *Medicine*, Dr. Jianbing Li's name appeared incorrectly as Jianbin Li.
